# *C*‐Reactive Protein–Triglyceride–Glucose Index and Coronary Artery Calcium Progression: A Prospective Cohort Analysis

**DOI:** 10.1002/clc.70304

**Published:** 2026-04-16

**Authors:** Qing‐Yun Hao, Ze‐Hua Li, Jun Weng, Zi‐Bin Zhan, Kun‐Hao Bai, Jing‐Bin Guo, Xiao‐Ping Cui, Jing‐Wei Gao, Yu‐Hong Zeng

**Affiliations:** ^1^ Department of Cardiology The affiliated Hospital of Inner Mongolia Medical University Hohhot China; ^2^ State Key Laboratory of Oncology in South China, Department of Endoscopy Sun Yat‐sen University Cancer Center, Guangdong Provincial Clinical Research Center for Cancer Guangzhou China; ^3^ Department of Hepatobiliary Surgery II, Zhujiang Hospital Southern Medical University Guangzhou China; ^4^ Laboratory of Heart Center, Department of Cardiology, Zhujiang Hospital Southern Medical University Guangzhou China; ^5^ Medical Engineering Department, Zhujiang Hospital Southern Medical University Guangzhou China; ^6^ Department of Cardiology, Sun Yat‐sen Memorial Hospital Sun Yat‐sen University Guangzhou China

**Keywords:** cardiovascular disease, coronary artery calcium, *C*‐reactive protein‐triglyceride‐glucose index, inflammation, insulin resistance, risk factors

## Abstract

**Objective:**

The *C*‐reactive protein–triglyceride–glucose index (CTI) reflects systemic inflammation and insulin resistance, but its relationship with coronary artery calcium (CAC) progression remains unclear. This study examined the association between CTI and CAC progression in a longitudinal cohort.

**Methods:**

Participants from the Coronary Artery Risk Development in Young Adults (CARDIA) study with CAC measurements at years 15, 20, and 25 were included. CTI was calculated from fasting blood samples at year 15 and categorized into quartiles. CAC was quantified using standardized computed tomography. CAC progression was defined as incident CAC > 0 for those with baseline CAC = 0, an annualized CAC increase ≥ 10 units for those with baseline CAC: 0–100, or an annualized percent increase ≥10% for those with baseline CAC ≥ 100. Cox proportional hazards models estimated hazard ratios (HRs), adjusting for cardiovascular risk factors.

**Results:**

Among 2655 participants (mean age 40.3 ± 3.6 years; 44.7% men), 704 (26.5%) experienced CAC progression over 8.9 ± 2.0 years. After adjustment, individuals in the highest CTI quartile had a 38% higher risk of CAC progression compared with the lowest quartile (HR = 1.380; 95% CI: 1.072–1.775). Subgroup analyses by age, sex, race, body mass index, and baseline CAC status were consistent, and results remained robust after excluding participants with baseline diabetes or lipid‐lowering medication use.

**Conclusions:**

Higher CTI was independently associated with increased CAC progression, supporting its potential utility as a biomarker for identifying individuals at elevated risk of subclinical atherosclerosis.

**Trial Registration:**

URL: https://www.clinicaltrials.gov; Unique identifier: NCT00005130.

## Graphical abstract

Higher CTI, reflecting inflammation and insulin resistance, was independently associated with greater coronary artery calcium progression in a longitudinal cohort. CTI may help identify individuals at risk for subclinical atherosclerosis.

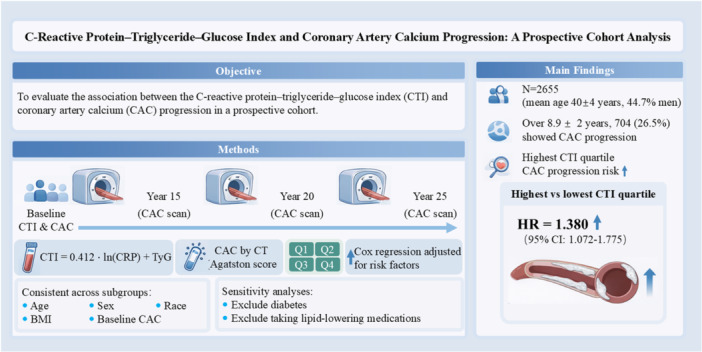

**Abbreviations**
BMI: body mass index
CAC: coronary artery calcium
CARDIA: Coronary Artery Risk Development in Young Adults
CI: Confidence Interval
CRP: *C*‐Reactive Protein
CT: computed tomography
CTI: *C*‐Reactive Protein–Triglyceride–Glucose Index
CVD: Cardiovascular Disease
HDL‐C: High‐Density Lipoprotein Cholesterol
HR: Hazard Ratio
IQR: Interquartile Range
LDL‐C: Low‐Density Lipoprotein Cholesterol
MDCT: Multidetector Computed Tomography
SD: Standard Deviation
VSMC: Vascular Smooth Muscle Cell

## 1 Introduction

Coronary artery calcium (CAC) is a hallmark of subclinical atherosclerosis and an established predictor of future cardiovascular events and mortality. It reflects the cumulative burden of atherosclerotic plaque and vascular injury and is widely used for cardiovascular risk stratification in asymptomatic individuals [1, 2, 3]. Identifying early biomarkers associated with CAC progression is critical for preventing adverse cardiovascular outcomes.

Insulin resistance (IR) and chronic low‐grade inflammation are two interrelated processes that have been independently associated with increased risk of CAC progression in large cohort studies. For example, the Multi‐Ethnic Study of Atherosclerosis (MESA) found that higher triglyceride‐glucoe (TyG) index, a surrogate marker for IR, was significantly associated with both prevalent and incident CAC [4]. This aligns with more recent evidence demonstrating that the TyG index predicts subclinical atherosclerosis and future cardiovascular events [5, 6]. Similarly, elevated levels of high‐sensitivity *C*‐reactive protein (hs‐CRP), a marker of systemic inflammation, have been shown to predict CAC progression and cardiovascular events in asymptomatic populations [7, 8, 9, 10]. Consistent with these findings, emerging studies have linked systemic inflammation to vascular calcification and atherosclerotic burden [11]. However, most existing studies have assessed IR and inflammation in isolation. The potential synergistic effect of these two processes on subclinical atherosclerosis—particularly CAC progression—has received limited attention. Given their shared contribution to vascular pathology, a composite biomarker that integrates both metabolic and inflammatory burden may offer greater prognostic value.

The *C*‐reactive protein‐triglyceride‐glucose (CTI) index is a novel composite biomarker that integrates *C*‐reactive protein (CRP), a sensitive marker of systemic inflammation, with the TyG index, a well‐validated surrogate for IR [12, 13]. CTI has recently shown prognostic value for stroke, coronary heart disease, and cancer mortality in population‐based studies [14, 15, 16]. More broadly, composite metabolic–inflammatory indices have demonstrated utility in cardiovascular risk prediction [17], and mechanistic studies support the interplay between inflammation, metabolic dysfunction, and endothelial injury in atherosclerosis [18]. Compared with CRP or TyG alone, CTI provides a more comprehensive assessment of the pro‐inflammatory and metabolic milieu underlying vascular damage [19]. However, evidence linking CTI to subclinical atherosclerosis, particularly to CAC progression, is currently limited.

To address this gap, we aimed to evaluate the prospective association between CTI and CAC progression using longitudinal data from the Coronary Artery Risk Development in Young Adults (CARDIA) study. We hypothesized that higher CTI levels would be independently associated with an increased risk of CAC progression, and that this relationship would remain robust across subgroups stratified by age, sex, race, body mass index (BMI), and baseline CAC status.

## 2 Methods

### 2.1 Study Design and Population

Details of the CARDIA study design have been previously described in depth elsewhere [20]. In brief, the CARDIA study is a prospective, multicenter cohort designed to investigate the evolution of cardiovascular disease (CVD) risk factors and outcomes over time. Between 1985 and 1986 (Year 0), the CARDIA study recruited 5115 participants, aged 18–30 years, consisting of Black and White men and women from four U.S. centers: Birmingham, Alabama; Chicago, Illinois; Minneapolis, Minnesota; and Oakland, California. Participants underwent follow‐up examinations at Years 2, 5, 7, 10, 15, 20, 25, and 30.

**Figure 1:**
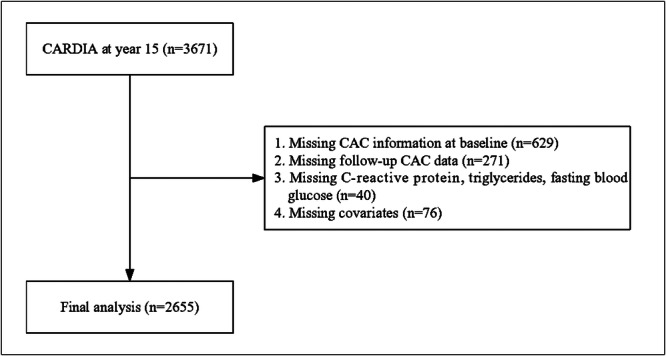
Flowchart for selection of study participants from the CARDIA cohort. Of the 3671 participants who completed the year 15 CARDIA examination, 1016 were excluded due to missing baseline CAC data (*n* = 629), missing follow‐up CAC data (*n* = 271), missing information on CRP, triglycerides, or fasting glucose (*n* = 40), or incomplete covariates (*n* = 76). A total of 2655 participants were included in the final analysis. CAC, coronary artery calcium; CARDIA, Coronary Artery Risk Development in Young Adults; CRP, *C*‐reactive protein.

CAC was first assessed using computed tomography (CT) scanning at Year 15 (2000–2001), representing the baseline CAC measurement for the present analysis. Of the 3671 participants who underwent CAC assessment at Year 15, individuals were excluded if they had missing baseline CAC data (*n* = 629), follow‐up CAC measurements (*n* = 271), missing data on CRP, triglycerides (TG), or fasting blood glucose (FPG) (*n* = 40), or incomplete covariate data (*n* = 76), as detailed in Figure 1. Thus, the final analytic sample included 2655 participants with complete datasets required for the current analysis.

### 2.2 Assessment of CTI

Blood samples were collected in the morning following a minimum of 8 h of overnight fasting. Samples were processed and analyzed in CARDIA‐certified central laboratories using standardized protocols. CRP, TG, and FPG concentrations were measured using well‐established enzymatic and immunoturbidimetric assays, as previously reported [21]. The CTI was calculated using the following formula: CTI = 0.412 × ln[CRP (mg/L)] + ln [TG (mg/dL) × FPG (mg/dL)/2]. Participants were categorized into quartiles according to their CTI values for subsequent analyses. Higher quartiles of CTI indicate greater systemic inflammation and higher levels of IR.

### 2.3 CAC Measurements

CAC was assessed at Years 15, 20, and 25 of the CARDIA study using standardized imaging protocols [22]. At Years 15 (2000–2001) and 20 (2005–2006), CAC was measured with either cardiac‐gated electron‐beam CT or multidetector CT (MDCT), depending on the field center. By Year 25 (2010–2011), all scans were performed exclusively with MDCT due to demonstrated high reproducibility. Prior studies have confirmed the equivalence of electron‐beam CT and multidetector CT, for CAC quantification [23]. All images were centrally read by trained technicians blinded to participants’ clinical data. CAC scoring was performed according to the Agatston method [24], which calculated lesion scores based on area and peak density. Scores for all calcified lesions within the left anterior descending, left main, circumflex, and right coronary arteries were summed to yield the total CAC score.

CAC progression was defined as meeting any of the following: 1) CAC > 0 at follow‐up among participants with baseline CAC = 0; 2) an annualized absolute increase in CAC ≥ 10 units among those with baseline CAC: 0–100; or 3) an annualized relative increase ≥10% (annualized change divided by baseline score) among those with baseline CAC ≥ 100 [25].

### 2.4 Measurements of Other Covariates

Demographic and lifestyle characteristics, including age, sex, race, and smoking status, were collected through structured, interviewer‐administered questionnaires. Smoking status was classified into three categories: current, former, or never smoker. Resting systolic and diastolic blood pressure measurements were obtained after participants rested in a seated position for at least 5 min. Blood pressure was measured three consecutive times on the right arm at 1 min intervals using a random‐zero sphygmomanometer. The mean value of the second and third measurements was recorded for analyses. Participants’ self‐reported physical activity was assessed via the validated CARDIA Physical Activity History questionnaire, administered by trained interviewers, as detailed previously [26]. Blood samples were drawn after overnight fasting (≥8 h) using EDTA‐containing vacutainer tubes. Plasma was immediately separated and stored at −70°C until transported to the central laboratory on dry ice. Laboratory assays, including total cholesterol, low‐density lipoprotein cholesterol (LDL‐C), high‐density lipoprotein cholesterol (HDL‐C), TG, CRP, serum creatinine, and FPG, were conducted following previously described standardized protocols [27]. Diabetes was defined as having FPG ≥ 126 mg/dL or self‐reported use of glucose‐lowering medications [28]. Hypertension was defined as either self‐reported diagnosis, use of antihypertensive medications, or having an average of three consecutive systolic blood pressure measurements ≥140 mmHg or diastolic blood pressure ≥90 mmHg [29]. BMI was calculated by dividing body weight (kg) by height squared (m²), with obesity defined as BMI ≥ 30 kg/m² according to common clinical guidelines [30].

### 2.5 Statistical Analysis

Data distribution was evaluated using the Shapiro–Wilk test. Continuous variables were presented as means ± standard deviations (SD) if normally distributed, or medians (interquartile ranges) if non‐normally distributed. Categorical variables were presented as counts and percentages. Differences between groups were assessed by one‐way ANOVA for normally distributed continuous variables, the Kruskal–Wallis *H* test for non‐normally distributed variables, and the *χ*² test for categorical variables.

The follow‐up period was defined from baseline at Year 15 (2000–2001) until the occurrence of CAC progression or until the end of Year 25 follow‐up (2010–2011), whichever occurred first. Kaplan‐Meier curves were generated to estimate the cumulative incidence of CAC progression stratified by CTI quartiles, with between‐group differences assessed using the log‐rank test. Cox proportional hazards regression models were employed to estimate hazard ratios (HRs) and corresponding 95% confidence intervals (CIs) for CAC progression across quartiles of CTI, using the lowest quartile as the reference. Multivariable Cox regression analyses were adjusted for key covariates, including age, sex, race, BMI, systolic blood pressure, diabetes mellitus, hypertension, LDL‐C, serum creatinine, smoking status, and physical activity.

Pre‐specified subgroup analyses were conducted according to age (≤ 41 or > 41 years), sex (male or female), race (White or Black), BMI (≤30 or >30 kg/m²), and baseline CAC status (presence or absence). Sensitivity analyses were performed by excluding participants with baseline diabetes or taking lipid‐lowering medications at baseline to evaluate potential confounding effects.

All statistical analyses were conducted using SPSS software (version 23.0, IBM Corp., Armonk, NY, USA) and R software (version 4.5.0). A two‐sided *p*‐value < 0.05 was considered statistically significant.

## 3 Results

### 3.1 Baseline Characteristics According to CTI Quartiles

The baseline demographic and clinical characteristics of participants stratified by quartiles of the CTI are summarized in Table 1. The mean age of the study population at baseline (Year 15) was 40.3 ± 3.6 years. Among the 2655 included participants, 1187 (44.7%) were male, 531 (20.0%) were current smokers, and 213 (8.0%) had detectable CAC at baseline. The overall mean CTI was 8.3 ± 0.7, with participants categorized into four quartiles: quartile 1 (Q1: mean CTI = 7.5 ± 0.2), quartile 2 (Q2: 8.1 ± 0.1), quartile 3 (Q3: 8.5 ± 0.1), and quartile 4 (Q4: 9.2 ± 0.4).

**Table 1:** Baseline characteristics of participants at year 15 by CTI status.

Characteristics	Total (*n* = 2655)	Q1 CTI (*n* = 664)	Q2 CTI (*n* = 664)	Q3 CTI (*n* = 664)	Q4 CTI (*n* = 663)	*p* value
Age, years	40.3 ± 3.6	40.1 ± 3.5	40.4 ± 3.6	40.4 ± 3.5	40.6 ± 3.7	0.093
Male, *n* (%)	1187 (44.7%)	236 (35.5%)	293 (44.1%)	309 (46.5%)	349 (52.6%)	< 0.001
White, *n* (%)	1491 (56.2%)	367 (55.3%)	393 (59.2%)	364 (54.8%)	367 (55.4%)	0.342
BMI, kg/m^2^	28.4 ± 6.0	25.1 ± 4.4	26.7 ± 4.9	29.4 ± 5.9	32.3 ± 6.1	< 0.001
Physical activity, exercise units	352.7 ± 279.6	388.1 ± 287.7	360.6 ± 275.5	351.3 ± 289.1	310.9 ± 260.3	< 0.001
SBP, mm Hg	112.5 ± 14.2	108.4 ± 13.4	110.8 ± 13.7	113.0 ± 13.9	117.6 ± 14.3	< 0.001
DBP, mm Hg	74.1 ± 11.1	71.3 ± 10.7	72.9 ± 10.7	75.0 ± 10.9	77.4 ± 11.1	< 0.001
Smoking status, *n* (%)						0.016
Never smoker	1629 (61.4%)	415 (62.5%)	423 (63.7%)	421 (63.4%)	370 (55.8%)	
Former smoker	495 (18.6%)	129 (19.4%)	113 (17%)	123 (18.5%)	130 (19.6%)	
Current smoker	531 (20.0%)	120 (18.1%)	128 (19.3%)	120 (18.1%)	163 (24.6%)	
Hypertension, n (%)	405 (15.3%)	57 (8.6%)	76 (11.4%)	116 (17.5%)	156 (23.5%)	< 0.001
Diabetes, n (%)	136 (5.1%)	643 (96.8%)	649 (97.7%)	637 (95.9%)	590 (89%)	< 0.001
Antihypertensive medication, n (%)	186 (7.0%)	14 (2.1%)	30 (4.5%)	60 (9%)	82 (12.4%)	< 0.001
LDL‐C, mg/dL	113.6 ± 31.6	101.8 ± 28.6	111.2 ± 29.1	117.8 ± 29.3	123.6 ± 34.7	< 0.001
HDL‐C, mg/dL	50.8 ± 14.4	59.8 ± 14.4	52.6 ± 12.8	48.4 ± 12.9	42.4 ± 11.6	< 0.001
TC, mg/dL	184.6 ± 34.1	172.0 ± 31.4	179.2 ± 30.0	186.8 ± 30.4	200.2 ± 37.6	< 0.001
TG, mg/dL	98.7 ± 59.5	49.9 ± 11.7	75.2 ± 16.9	100.8 ± 29.4	169.0 ± 70.7	< 0.001
Fasting glucose, mg/dL	85.8 ± 17.9	79.9 ± 7.8	82.4 ± 8.2	85.6 ± 11.4	95.4 ± 29.7	< 0.001
Serum creatinine, mg/dL	1.0 ± 0.2	1.0 ± 0.2	1.0 ± 0.3	1.0 ± 0.2	1.0 ± 0.2	0.002
*C* reactive protein, mg/dL	2.0 ± 2.6	0.9 ± 0.4	1.3 ± 1.0	2.1 ± 1.8	3.6 ± 4.4	< 0.001
CTI	8.3 ± 0.7	7.5 ± 0.2	8.1 ± 0.1	8.5 ± 0.1	9.2 ± 0.4	< 0.001
Baseline coronary artery calcium, *n* (%)	213 (8.9%)	34 (5.1%)	58 (8.7%)	66 (9.9%)	84 (12.7%)	< 0.001

Abbreviations: BMI, body mass index; CTI, *C*‐reactive protein‐triglyceride glucose index; DBP, diastolic blood pressure; HDL‐C, high density lipoprotein cholesterol; LDL‐C, low density lipoprotein cholesterol; SBP, systolic blood pressure; TC, total cholesterol; TG, triglycerides.

Participants in the highest quartile of CTI (Q4) were more likely to be male and current smokers. Additionally, these participants exhibited significantly higher BMI, systolic and diastolic blood pressures, FPG, TG, LDL‐C, total cholesterol, and CRP levels. They also had a higher prevalence of hypertension, but lower HDL‐C and lower physical activity levels compared to those in the lower quartiles. The prevalence of diabetes was also lower in participants within the highest CTI quartile. No statistically significant differences were observed among quartile groups regarding age or racial distribution.

### 3.2 Association Between CTI and CAC Progression

**Figure 2:**
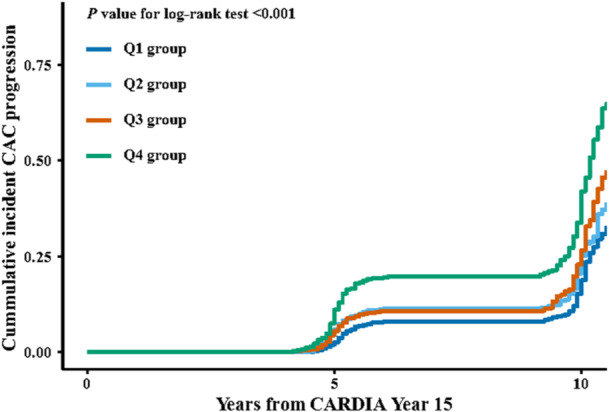
Cumulative incidence of coronary artery calcium (CAC) progression by CTI quartiles in the CARDIA (Coronary Artery Risk Development in Young Adults) study. Kaplan‐Meier curves show significantly different cumulative incidence of CAC progression across quartiles of the *C*‐reactive protein–triglyceride glucose index (CTI) (log‐rank *p* < 0.001). Participants in the highest CTI quartile (Q4) had the greatest incidence of CAC progression, while those in the lowest quartile (Q1) had the lowest incidence.

During a mean follow‐up of 8.9 ± 2.0 years, 704 participants (26.5%) experienced CAC progression. Figure 2 shows the cumulative incidence of CAC progression stratified by CTI quartile, with significantly higher progression observed in the upper quartiles (log‐rank *p* < 0.001).

Multivariable‐adjusted Cox regression analysis (Table 2) revealed a significant association between higher CTI and greater risk of CAC progression. Compared with Q1, participants in Q4 had a 38.0% higher risk of CAC progression (HR: 1.380; 95% CI: 1.072–1.775; *p*  <  0.05), after adjusting for demographics, lifestyle, comorbidities, and clinical parameters.

**Table 2:** Risk of CAC progression for baseline CTI levels.

Groups	No. Events/total	Model 1 HR (95% CI)	*p* value	Model 2 HR (95% CI)	*p* value	Model 3 HR (95% CI)	*p* value
Q1	115/664	Reference	1.0	Reference	1.0	Reference	1.0
Q2	149/664	1.332 (1.044–1.699)	0.021	1.204 (0.944–1.537)	0.135	1.064 (0.832–1.361)	0.620
Q3	175/664	1.570 (1.241–1.987)	< 0.001	1.383 (1.092–1.751)	0.007	1.100 (0.861–1.407)	0.445
Q4	265/663	2.585 (2.076–3.218)	< 0.001	2.177 (1.746–2.715)	< 0.001	1.380 (1.072–1.775)	0.012

*Note:* Model 1: Unadjusted.

Model 2: Adjusted for age, race, and sex.

Model 3: Adjusted for model 2 covariates plus body mass index, diabetes, hypertension, low density lipoprotein cholesterol, physical activity, serum creatinine, smoking status and systolic blood pressure.

Abbreviations: CAC, coronary artery calcium; CI, confidence interval; CTI, *C*‐reactive protein‐triglyceride glucose index; HR, hazard ratio.

In additional analyses, we replaced CTI with its individual components, CRP and the TyG index, in the fully adjusted Cox models. Higher CRP and higher TyG index were each associated with increased risk of CAC progression; however, the effect estimates were weaker compared with those observed for CTI. These comparative results are summarized in Tables S1 and S2.

### 3.3 Subgroup and Sensitivity Analysis

**Figure 3:**
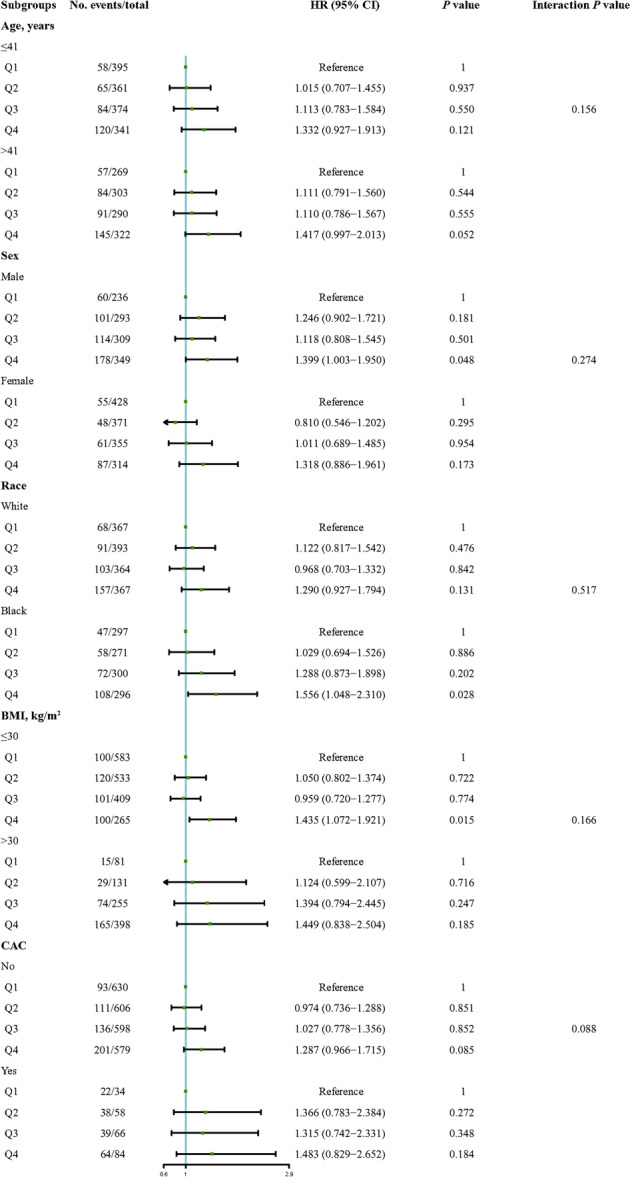
Adjusted subgroup analysis of the association between CTI (*C*‐reactive protein–triglyceride glucose index) and coronary artery calcium (CAC) progression. Cox regression analyses were adjusted for age, race, sex, body mass index (BMI), systolic blood pressure (SBP), low‐density lipoprotein cholesterol (LDL‐C), diabetes status, smoking, and total energy intake. Subgroup analyses were stratified by age, sex, race, BMI, and baseline CAC status. Hazard ratios (HRs) and 95% confidence intervals (CIs) were derived using the lowest CTI quartile as the reference in each subgroup.

As shown in Figure 3, stratified subgroup analyses by age (<41 or ≥41 years), sex (male or female), race (White or Black), BMI (<30 or ≥30 kg/m²), and baseline CAC status (present or absent) consistently demonstrated a positive association between CTI and CAC progression, with no significant interaction effects.

In sensitivity analyses, we first excluded participants with baseline diabetes (Table S3), and then further excluded those taking lipid‐lowering medications at baseline (Table S4). In both analyses, the association between CTI and CAC progression remained robust, indicating that the observed relationship is not explained solely by the presence of diabetes or the use of lipid‐lowering therapy.

## 4 Discussion

In this longitudinal community‐based cohort study from the CARDIA project with an average follow‐up of 8.9 ± 2.0 years, we identified several key findings: (1) higher CTI levels in early midlife were independently associated with an increased risk of CAC progression in midlife; and (2) this relationship was consistently observed across stratified subgroups and remained robust after excluding participants with baseline diabetes or taking lipid‐lowering medications at baseline. Importantly, additional comparative analyses demonstrated that CTI exhibited a stronger association with CAC progression than its individual components, CRP and the TyG index.

CAC progression reflects ongoing subclinical atherosclerosis and is a well‐established surrogate marker for future cardiovascular events and mortality [31, 32]. Identifying upstream risk markers during the preclinical phase is essential for preventive strategies. While IR and chronic low‐grade inflammation have long been recognized as critical contributors to atherogenesis and vascular calcification [33, 34, 35, 36, 37], most prior studies have examined these two processes in isolation. For instance, an elevated TyG index has been associated with both the presence and progression of CAC in cohorts such as MESA and the Korean Genome and Epidemiology Study [13, 34], while hs‐CRP has independently predicted CAC burden and cardiovascular events in asymptomatic populations [38]. In contrast, our findings indicate that integrating inflammatory and metabolic information into a single composite index offers added value beyond assessing either pathway alone. In fully adjusted models, CTI outperformed CRP and the TyG index individually with respect to the strength of association with CAC progression, supporting its incremental predictive utility.

What distinguishes our study is the integrated evaluation of these two pathways through the CTI—a composite biomarker that merges inflammatory and metabolic risk into a single metric. Although recent studies have reported associations between CTI and major adverse cardiovascular events, stroke, and cancer mortality [39, 40, 41], evidence linking CTI to subclinical atherosclerosis, particularly CAC progression, has been lacking. To our knowledge, this is the first study to demonstrate a significant prospective association between CTI and CAC progression in a general population, and to directly show that this association is stronger than those observed for CRP or the TyG index alone, independent of traditional cardiovascular risk factors such as SBP, LDL‐C, diabetes, and smoking. While these associations were statistically robust, the magnitude of effect was modest, suggesting that CTI should be interpreted as an incremental rather than deterministic risk indicator in the context of CAC progression.

Importantly, no significant interaction was observed between CTI and demographic or clinical characteristics, including age, sex, race, BMI, or baseline CAC status. This suggests that CTI has potential generalizability as a risk stratification tool across diverse subpopulations. In addition, sensitivity analyses excluding participants with baseline diabetes or taking lipid‐lowering medications at baseline yielded consistent results, reinforcing the independence of the CTI–CAC progression association from overt metabolic disease.

Nevertheless, the performance of CTI may differ across patient populations with varying metabolic and inflammatory profiles. As a composite inflammatory–metabolic index, CTI may be particularly informative in individuals with higher metabolic burden or low‐grade chronic inflammation—such as those with obesity, insulin resistance, or early cardiometabolic dysfunction—where inflammatory–metabolic dysregulation plays a central role in atherosclerotic progression. In contrast, among individuals with very high baseline cardiovascular risk or more advanced calcification, traditional risk factors and structural disease burden may dominate risk prediction, potentially attenuating the incremental contribution of CTI. Moreover, demographic factors such as age, sex, and race may influence the relative contribution of CTI components, given known differences in inflammatory responses, insulin sensitivity, and lipid metabolism across these groups. Although our subgroup analyses demonstrated generally consistent associations across major demographic and clinical strata, these considerations highlight the importance of further studies to evaluate population‐specific performance and to determine whether tailored application strategies could enhance the clinical utility of CTI.

Mechanistically, chronic inflammation and IR are known to act synergistically to promote vascular injury. Elevated CRP levels signal heightened inflammatory activity, while TyG reflects impaired glucolipid metabolism. Together, they contribute to oxidative stress, reduced nitric oxide bioavailability, and endothelial dysfunction [42, 43]. These conditions facilitate the activation of pro‐calcific signaling pathways such as NF‐κB and BMP2, driving vascular smooth muscle cell osteogenic switching and calcium deposition [33]. IR may further aggravate this process through increased lipid accumulation, cellular apoptosis, and microvascular rarefaction. By jointly capturing these interrelated processes, CTI may better reflect the underlying metabolic–inflammatory milieu driving early atherogenesis than either component considered in isolation. Thus, CTI may represent an integrative risk indicator capturing the metabolic‐inflammatory axis involved in early atherogenesis and vascular calcification.

From a clinical perspective, CTI is not intended to serve as a diagnostic threshold but rather as a relative risk stratification marker. In the absence of universally accepted cutoff values, we analyzed CTI in quartiles to characterize graded risk associations and to minimize reliance on cohort‐specific thresholds. This approach aligns with the exploratory evaluation of emerging composite biomarkers in population‐based studies. Importantly, CTI may have potential utility as a complementary marker alongside established cardiovascular risk assessment tools, such as the ASCVD risk calculator. In particular, CTI could help refine risk stratification in individuals classified as having intermediate or discordant risk based on traditional factors, by capturing additional inflammatory–metabolic burden not fully reflected in existing models. Given the modest effect sizes observed in our study, clinical translation will require careful evaluation of the incremental benefit of CTI beyond standard risk factors. Formal validation in independent cohorts with diverse demographic and clinical characteristics will be necessary before considering routine clinical implementation or integration into risk prediction algorithms.

While the CARDIA cohort offers a rare opportunity to examine CAC development beginning in young adulthood, the relatively short follow‐up duration (mean 8.9 years) may not fully capture the entire lifetime trajectory of coronary calcification. CAC often accelerates later in adulthood, and younger participants without calcification during early to midlife may still develop CAC at older ages. Therefore, our findings primarily reflect early to midlife CAC progression rather than long‐term or late‐onset calcification. Nevertheless, repeated standardized CT assessments in CARDIA allow for the characterization of early subclinical atherosclerosis during a critical developmental window, providing clinically relevant insight into risk pathways preceding overt cardiovascular disease. Future work involving extended follow‐up of this cohort and validation in older populations will be essential to determine whether the observed associations persist across later life stages and fully capture the spectrum of CAC progression.

Our study benefits from several strengths: a well‐characterized biracial cohort, standardized assessment of CAC progression, and long‐term follow‐up. However, some limitations should be noted. First, as an observational study, we cannot establish causality. Second, CTI was derived from a single time point, which may not fully capture the dynamic nature of systemic inflammation and metabolic dysfunction. Third, although the associations were statistically significant, the clinical significance may be modest given the effect sizes, underscoring the need for external validation. Fourth, finally, the CARDIA cohort primarily includes Black and White young adults, which may limit the generalizability of our findings to other populations.

In conclusion, our findings suggest that CTI is a novel and independent predictor of CAC progression in middle‐aged adults. Beyond its individual components, CTI provides incremental predictive information for subclinical coronary atherosclerosis progression, highlighting the value of integrating inflammatory and metabolic risk pathways. Given the modest effect sizes and the need for external validation, CTI should be viewed as an incremental risk indicator rather than a stand‐alone determinant of CAC progression. When interpreted as a relative risk indicator and used in conjunction with established risk assessment frameworks, CTI may offer a practical approach to identifying individuals at elevated risk of subclinical coronary atherosclerosis. Future studies are needed to validate these findings in other populations and to explore whether interventions targeting systemic inflammation and IR can attenuate CTI levels and reduce CAC burden.

## Author Contributions

Yu‐Hong Zeng conceptualized the study. Qing‐Yun Hao and Jun Weng conducted the statistical analysis. Jing‐Wei Gao provided substantive expertise and revised the manuscript. Ze‐Hua Li drafted the manuscript. Zi‐Bin Zhan, Jing‐Bin Guo, and Xiao‐Ping Cui contributed expertise on statistical analysis. Qing‐Yun Hao, Ze‐Hua Li, Zi‐Bin Zhan, Jing‐Bin Guo, Xiao‐Ping Cui, Kun‐Hao Bai, Jing‐Wei Gao, and Yu‐Hong Zeng critically reviewed the manuscript. Jing‐Wei Gao further revised the manuscript. Yu‐Hong Zeng is responsible for the final content of the manuscript. All authors read and approved the final manuscript.

## Conflicts of Interest

The authors declare no conflicts of interest.

## Supporting information

Supporting File

## Data Availability

Data described in the manuscript, code book, and analytic code will be made available upon request, pending application and approval.
