# Supplementary material for: C‐Reactive Protein–Triglyceride–Glucose Index and Coronary Artery Calcium Progression: A Prospective Cohort Analysis
Source: Clin Cardiol. 2026 Apr 16;49(4):e70304. doi: 10.1002/clc.70304 (PMC13085215; doi:10.1002/clc.70304)
Supplement: Supplementary file 1 — Supporting File [file CLC-49-e70304-s001.docx]

**Table S1 Risk of CAC progression for baseline CRP**

| **Groups** | **No. Events/total** | **Model 1 HR (95% CI)** | ***P* value** | **Model 2 HR (95% CI)** | ***P* value** | **Model 3 HR (95% CI)** | ***P* value** |
| --- | --- | --- | --- | --- | --- | --- | --- |
| Q1 | 166/664 | Reference | 1.0 | Reference | 1.0 | Reference | 1.0 |
| Q2 | 181/664 | 1.079 (0.874-1.332) | 0.482 | 1.104 (0.894-1.363) | 0.359 | 1.058 (0.856-1.308) | 0.601 |
| Q3 | 156/664 | 0.925 (0.743-1.151) | 0.484 | 1.075 (0.863-1.340) | 0.517 | 0.958 (0.767-1.197) | 0.707 |
| Q4 | 201/663 | 1.294 (1.054-1.590) | 0.014 | 1.739 (1.407-2.149) | <0.001 | 1.185 (0.936-1.499) | 0.159 |

Model 1: Unadjusted.

Model 2: Adjusted for age, race and sex.

Model 3: Adjusted for model 2 covariates plus body mass index, diabetes, hypertension, low density lipoprotein cholesterol, physical activity, serum creatinine, smoking status and systolic blood pressure.

CTI, C-reactive protein-triglyceride glucose index; CAC, coronary artery calcium; CI, confidence interval; HR, hazard ratio.

**Table S2 Risk of CAC progression for baseline TyG**

| **Groups** | **No. Events/total** | **Model 1 HR (95% CI)** | ***P* value** | **Model 2 HR (95% CI)** | ***P* value** | **Model 3 HR (95% CI)** | ***P* value** |
| --- | --- | --- | --- | --- | --- | --- | --- |
| Q1 | 103/664 | Reference | 1.0 | Reference | 1.0 | Reference | 1.0 |
| Q2 | 151/664 | 1.484 (1.155-1.907) | 0.002 | 1.328 (1.033-1.708) | 0.027 | 1.118 (0.867-1.441) | 0.391 |
| Q3 | 189/664 | 1.927 (1.514-2.451) | <0.001 | 1.615 (1.266-2.060) | <0.001 | 1.266 (0.984-1.628) | 0.066 |
| Q4 | 264/663 | 2.795 (2.225-3.510) | <0.001 | 2.064 (1.633-2.609) | <0.001 | 1.271 (0.983-1.644) | 0.067 |

Model 1: Unadjusted.

Model 2: Adjusted for age, race and sex.

Model 3: Adjusted for model 2 covariates plus body mass index, diabetes, hypertension, low density lipoprotein cholesterol, physical activity, serum creatinine, smoking status and systolic blood pressure.

CTI, C-reactive protein-triglyceride glucose index; CAC, coronary artery calcium; CI, confidence interval; HR, hazard ratio.

**Table S3 Risk of CAC progression for baseline CTI in participants without baseline diabetes**

| **Groups** | **No. Events/total** | **Model 1 HR (95% CI)** | ***P* value** | **Model 2 HR (95% CI)** | ***P* value** | **Model 3 HR (95% CI)** | ***P* value** |
| --- | --- | --- | --- | --- | --- | --- | --- |
| Q1 | 110/643 | Reference | 1.0 | Reference | 1.0 | Reference | 1.0 |
| Q2 | 145/649 | 1.350 (1.054-1.730) | 0.018 | 1.227 (0.957-1.573) | 0.107 | 1.072 (0.835-1.377) | 0.586 |
| Q3 | 164/637 | 1.553 (1.220-1.977) | <0.001 | 1.366 (1.071-1.741) | 0.012 | 1.069 (0.831-1.376) | 0.604 |
| Q4 | 236/590 | 2.626 (2.094-3.294) | <0.001 | 2.175 (1.730-2.733) | <0.001 | 1.378 (1.065-1.784) | 0.015 |

Model 1: Unadjusted.

Model 2: Adjusted for age, race and sex.

Model 3: Adjusted for model 2 covariates plus body mass index, diabetes, hypertension, low density lipoprotein cholesterol, physical activity, serum creatinine, smoking status and systolic blood pressure.

CTI, C-reactive protein-triglyceride glucose index; CAC, coronary artery calcium; CI, confidence interval; HR, hazard ratio.

**Table S4 Risk of CAC progression for baseline CTI in participants not taking lipid-lowering medications at baseline**

| **Groups** | **No. Events/total** | **Model 1 HR (95% CI)** | ***P* value** | **Model 2 HR (95% CI)** | ***P* value** | **Model 3 HR (95% CI)** | ***P* value** |
| --- | --- | --- | --- | --- | --- | --- | --- |
| Q1 | 110/638 | Reference | 1.0 | Reference | 1.0 | Reference | 1.0 |
| Q2 | 135/635 | 1.280 (0.994-1.647) | 0.056 | 1.169 (0.907-1.505) | 0.227 | 1.016 (0.788-1.312) | 0.901 |
| Q3 | 155/624 | 1.501 (1.175-1.918) | 0.001 | 1.332 (1.041-1.703) | 0.022 | 1.029 (0.796-1.330) | 0.827 |
| Q4 | 229/577 | 2.607 (2.075-3.275) | <0.001 | 2.182 (1.733-2.746) | <0.001 | 1.364 (1.050-1.773) | 0.020 |

Model 1: Unadjusted.

Model 2: Adjusted for age, race and sex.

Model 3: Adjusted for model 2 covariates plus body mass index, diabetes, hypertension, low density lipoprotein cholesterol, physical activity, serum creatinine, smoking status and systolic blood pressure.

CTI, C-reactive protein-triglyceride glucose index; CAC, coronary artery calcium; CI, confidence interval; HR, hazard ratio.
